# The Proportion of Chromatin Graded between Closed and Open States Determines the Level of Transcripts Derived from Distinct Promoters in the *CYP19* Gene

**DOI:** 10.1371/journal.pone.0128282

**Published:** 2015-05-28

**Authors:** Naoe Kotomura, Nobuhiro Harada, Satoru Ishihara

**Affiliations:** Department of Biochemistry, Fujita Health University School of Medicine, Toyoake, Aichi, Japan; South Texas Veterans Health Care System and University of Texas Health Science Center at San Antonio, UNITED STATES

## Abstract

The human *CYP19* gene encodes aromatase, which converts androgens to estrogens. *CYP19* mRNA variants are transcribed mainly from three promoters. Quantitative RT-PCR was used to measure the relative amounts of each of the three transcripts and determine the on/off state of the promoters. While some of the promoters were silent, *CYP19* mRNA production differed among the other promoters, whose estimated transcription levels were 0.001% to 0.1% of that of the *TUBB* control gene. To investigate the structural aspects of chromatin that were responsible for this wide range of activity of the *CYP19* promoters, we used a fractionation protocol, designated SEVENS, which sequentially separates densely packed nucleosomes from dispersed nucleosomes. The fractional distribution of each inactive promoter showed a similar pattern to that of the repressed reference loci; the inactive regions were distributed toward lower fractions, in which closed chromatin comprising packed nucleosomes was enriched. In contrast, active *CYP19* promoters were raised toward upper fractions, including dispersed nucleosomes in open chromatin. Importantly, these active promoters were moderately enriched in the upper fractions as compared to active reference loci, such as the *TUBB* promoter; the proportion of open chromatin appeared to be positively correlated to the promoter strength. These results, together with ectopic transcription accompanied by an increase in the proportion of open chromatin in cells treated with an H3K27me inhibitor, indicate that *CYP19* mRNA could be transcribed from a promoter in which chromatin is shifted toward an open state in the equilibrium between closed and open chromatin.

## Introduction

Transcription is often defined as the first regulatable step in gene expression, and in this step a specific gene (or set of genes) is targeted within the genome. In this process, transcription factors (TFs) must bind to regulatory sequences of target genes [1,2], and each gene requires an individual combination of TFs for its activation. Importantly, most TFs have no enzymatic activity, but each functions as an adaptor molecule for secondary proteins called co-activators or co-repressors [3]. These secondary proteins act as enzymes that change the environment of the promoter region and eventually regulate whether or not the promoter is activated. Such changes are called chromatin remodeling and fall into two classes: movements of nucleosomes and covalent modifications of histone molecules [4,5].

DNA in cells is packed into chromatin, and the primary components of chromatin are nucleosomes that comprise eight histone proteins. Because this structure usually becomes an obstacle for events that occur on the DNA, the removal of nucleosomes from a promoter region must precede transcription [4–6]. In fact, the number of nucleosomes at an inducible promoter decreases upon initiation of transcription [7–9]. Moreover, genome-wide analyses have revealed that nucleosome-depleted regions (NDRs) are evident around transcription start sites (TSSs) of highly expressed genes [10–12]. During nucleosome removal or repositioning, a chromatin-remodeling ATPase catalyzes the sliding of a nucleosome along DNA [13]. This kind of enzyme is recruited by regular TFs to target promoters; for example, p53 can recruit SMARCA4 (also known as BRG1) to the *CDKN1A* (p21) promoter [14]. Other TFs help recruit enzymes that introduce or remove histone modifications; such modifications also have a big impact on chromatin structure. For example, when lysine residues of histones become acetylated, nucleosomes comprising these acetylated histones lose affinity for DNA; consequently, the chromatin structure loosens, and a respective promoter becomes more accessible [15]. This type of structural alteration occurs at activated promoters following TF-mediated recruitment of histone acetyltransferase (HAT). A well-characterized HAT, CBP/p300, is recruited to a promoter sequence by various TFs such as CREB, a TF binding to a cAMP-responsive element [16]. Conversely, histone deacetylases (HDACs) that remove acetyl groups from histone can also be recruited to chromatin by TFs [17]. In addition, histone modifications triggered by the binding of TFs are used as a recognition site for tertiary proteins, e.g. bromodomain and chromodomain proteins that recognize acetylated and methylated histones, respectively; these tertiary proteins also influence chromatin structure [18,19]. These findings indicate that TFs alter chromatin structure through a combination of histone modifications. Although an individual TF is usually categorized as a positive or a negative regulator of transcription based on its binding partner(s), chromatin structure when affected by a combination of TFs is not simply divided into closed or open chromatin. A recent statistical analysis indicates that the level of expression of a gene is related to the binding of TFs [20]. This finding suggests that chromatin structure at physically distinct promoters that are combinatorially affected by multiple TFs could be responsible for the transcription level, namely the strength of a respective promoter.

The human *CYP19* gene encodes aromatase, an enzyme that converts androgens to estrogens, and is expressed exclusively in a set of steroidogenic tissues [21]. Transcription of *CYP19* mRNA initiates at multiple promoters that each result in a distinct mRNA consisting of a distinct noncoding first exon and common coding downstream exons. Reportedly, the promoter usage differs among cell types that express this gene [22,23]. Moreover, this usage is changed in breast adipose tissue as the total amount of *CYP19* transcripts increases along with the development of breast cancer, indicating that the transcription level could be controlled by the promoter usage [24]. Here, to investigate a relationship between the transcriptional state and the chromatin structure of promoters with different activity, we compared the activity of *CYP19* promoters in three human cell lines in which the *CYP19* gene is transcribed or not transcribed.

## Materials and Methods

### Cell culture

HepG2, JEG-3, and HeLa cells were cultured in Minimum Essential Medium with alpha modification supplemented with 10% calf serum and antibiotics. KGN cells were cultured in Dulbecco's Modified Eagle Medium: Nutrient Mixture F-12 supplemented with 10% fetal bovine serum and antibiotics. To study the contribution of H3K27me3 to transcription of the *CYP19* gene, the cells were harvested following culture for 3 days with 10 μM of 3-deazaneplanocin A (DZNep; Cayman Chemical or BioVision).

### RT-PCR

RNeasy micro spin columns (Qiagen) were used to prepare total RNA from cells. Superscript III Reverse Transcriptase and random primers (Life Technologies) were used to synthesize cDNA from total RNA; the cDNA was then purified and concentrated on a MinElute spin column (Qiagen) following treatment with RNase A plus RNase H and extraction with phenol/chloroform. For conventional PCR, 2 ng of cDNA template, which was quantified with a Quant-iT OliGreen Kit (Life Technologies), was used in each single reaction. Each PCR comprised 34 cycles (except for the Ia transcripts in the assay with DZNep; 40 cycles) with a primer set capable of amplifying a region derived from two separate exons. Each amplicon was size-fractionated in a 4% agarose gel to determine whether it was of the correct size. For quantitative PCR, each cDNA sample diluted from 0.0004 to 10 ng was used for a single reaction. To make a standard curve for comparison between PCR signals amplified with a different primer set, serially diluted human genomic DNA (0.76–12,500 pg) was utilized as described previously [25]. Primer sets were designed to amplify a region within a single exon. The amount of each transcript was estimated from the respective PCR cycle threshold (Ct) value plotted on the standard curve. Each PCR reaction was performed by using a 1:3 mixture of a QuantiFast SYBR Green PCR Kit (Qiagen) and a FastStart SYBR Green Master (Roche) in a real-time PCR machine (Applied Biosystems 7900HT, 7300, or 7000; Life Technologies). The primers used in this study are listed in S1 Table.

### Flow cytometry

Three to 8 million cells were dispersed following treatment with PBS supplemented with 0.25% trypsin. The harvested cells were then crosslinked with 3.7% formaldehyde in PBS for 10 min at 37°C and permeabilized for 30 min on ice in nine volumes of ice-cold methanol. After being washed with PBS twice, the cells were re-suspended in PBS supplemented with 2 mg/ml BSA. Finally, the cells were incubated with primary antibody for 1 hr at room temperature and then with secondary antibody for an additional 30 min at room temperature. The stained cells were analyzed by using a FACScan flow cytometry analyzer (BD). The primary antibodies were used as follows: anti-aromatase (*CYP19* product), #SM2222PS, Acris; mouse IgG2a isotype control, #M076-3, MBL. Alexa Fluor 488-conjugated anti-mouse IgG Fab fragment (Life Technologies) was used for the secondary antibody.

### The Sedimentation velocity method followed by normalization in the size of the DNA (SEVENS) assay

The principle of the SEVENS assay has been described previously [26]. The published protocol for this assay was modified as follows. Cells (10 to 15 mg wet weight) were collected by centrifugation and then incubated at room temperature for 10 min in PBS supplemented with 0.75% formaldehyde, which was quenched by incubating with 125 mM glycine. The formaldehyde-treated cells were washed in ice-cold PBS twice, suspended in 250 μl of SDS lysis buffer (1% SDS, 50 mM Tris-HCl (pH 8.0), 10 mM EDTA, and a Complete Protease Inhibitor Cocktail (Roche)), and incubated at 37°C for 2 hrs. A Branson Sonifier 150 set at level 2 was then used to disrupt the cells via two 5-sec sonication treatments. Each resulting lysate was spun down at 14,000 x g for 10 min to remove debris and layered onto a 9.6 ml sucrose gradient (6–15%) in 1.1% Triton X-100, 0.01% SDS, 16.7 mM Tris-HCl (pH 8.0), 1.2 mM EDTA, 167 mM NaCl, and a Complete Protease Inhibitor Cocktail (Roche) in a polyallomer centrifugation tube (Beckman Coulter). Each sample was then subjected to ultracentrifugation at 256,000 x g for 4 hrs at 4°C by using a Beckman swing rotor SW41Ti. We collected twelve 0.8-ml fractions from each gradient by sequentially removing layers of sample from the top to the bottom of each tube with a micropipette; each 0.8-ml fraction was heated at 65°C overnight to reverse the crosslinking. After treatment with RNase A and Proteinase K, the DNA in each fraction was purified by phenol/chloroform extraction and subsequent 2-propanol precipitation. The DNA recovered from each fraction was loaded onto a 1.5% low-melting-point agarose gel in a Tris–acetate–EDTA buffer, and the gel was cut to collect DNA ranging in size from 0.4 to 0.6 kb. A MinElute spin column (Qiagen) was utilized to purify the DNA from the gel block. The 12 DNA preparations normalized with regard to size were quantified with a Quant-iT PicoGreen Kit (Life Technologies) and then divided into two merged preparations by the amount of the DNA in the upper (Up) and the lower (Lo) halves of the fractions. A 250 pg sample of each half was applied to quantitative PCR as described above to estimate the fold enrichment in the upper half relative to the lower half (Up/Lo), which was plotted as a log2 value on the x-axis showing the *CYP19* locus. Another SEVENS assay, in which six 1.6-ml fractions were collected, was also performed to analyze the fractional distribution of a given sequence. Following quantitative PCR for each fraction, the enrichment of a given sequence in each fraction relative to in whole merged fractions was calculated. PCR primers used in the assays are listed in S1 Table.

### Chromatin immunoprecipitation (ChIP) assay

Chromatin crosslinked in the two-step method [27] was subjected to a ChIP assay as described previously [25]. An aliquot containing 1 to 5 μg of DNA (estimated by using a Quant-iT PicoGreen Kit (Life Technologies)) was used for a single ChIP reaction. The primary antibodies were used as follows: anti-pan-histone H3, #ab1791, Abcam; anti-H3K27ac, #39685, Active Motif; anti-H3K27me3, #9733, Cell Signaling. Immunoprecipitation of protein-DNA complexes was performed with secondary antibody-conjugated Dynabeads M-280 (Life Technologies). To detect co-precipitated DNA, quantitative PCR was performed as described above. PCR primers used for these assays are listed in S1 Table.

### Western blotting

The cultured cells were suspended in western lysis buffer (55 mM Tris-HCl (pH 6.8), 0.72 M 2-mercaptoethanol, 2% SDS, 0.005% bromophenol blue, and 8% glycerol) and boiled for 5 min. After centrifugation at 14,000 xg for 5 min, each supernatant was collected as a cell lysate. An EZQ Protein Quantitation Reagent (Life Technologies) was used to measure the protein concentration of each sample. Each protein sample (5 to 10 μg) was loaded onto a SDS-PAGE mini-gel; separated proteins were then blotted onto a polyvinylidene difluoride membrane (Merck Millipore). After sequential exposures to a primary antibody, a biotinylated secondary antibody (GE Healthcare), and a streptavidin-conjugated alkaline phosphatase (GE Healthcare), each membrane was developed with Western Blue (Promega). The primary antibodies were used as follows: anti-aromatase (*CYP19* product), #SM2222PS, Acris; anti-β-actin, #A1978, Sigma; anti-pan-histone H3, #ab1791, Abcam; anti-H3K27me3, #9733, Cell Signaling; anti-EZH2, #5246, Cell Signaling.

### Statistical analysis

Statistical analysis was performed with Microsoft Excel. Each numeric data point was calculated from at least three independent measurements and is represented as mean ± SE. The p-values were generated using Student’s t-test.

## Results

### Assessment of transcription from three promoters in the human *CYP19* gene

**Fig 1:**
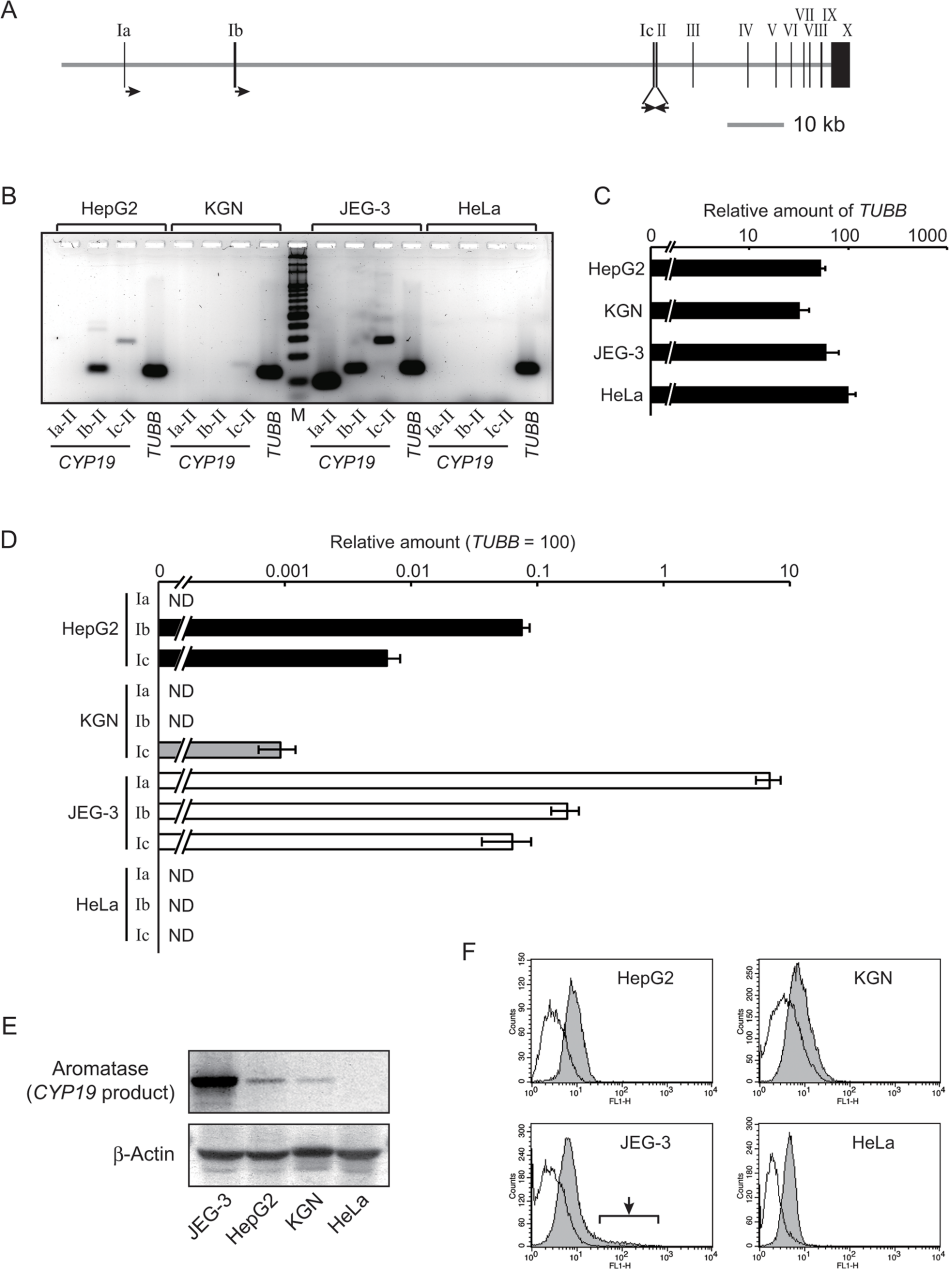
Transcription from each of three *CYP19* promoters. (A) The gene structure of the human *CYP19* locus. Closed boxes with Roman numerals represent exons of the gene. Primers for conventional RT-PCR are denoted as arrows; the three rightward arrows are forward primers for the respective first exons, while the leftward arrow is a reverse primer for the common second exon. (B) Conventional RT-PCR analyses revealed the existence of three kinds of *CYP19* transcripts. “Ia-II”, “Ib-II”, or “Ic-II” indicates a PCR reaction amplified with a primer set annealing to exons Ia/II, exons Ib/II, or exons Ic/II, respectively. PCR for the “*TUBB*” (β-tubulin) gene was performed as a control. “M” denotes a molecular weight marker. (C) Quantitative RT-PCR analyses revealed a comparable level of the *TUBB* transcripts among HepG2, KGN, and JEG-3 cell lines following normalization to the amount of 18S rRNA. The quantitative similarity was confirmed after performing Student’s t-test (p > 0.05). The value for the transcripts in the three cell lines is presented as a percentage of that in HeLa cells. (D) The relative amount of each kind of the *CYP19* transcript was estimated by using quantitative RT-PCR (see Materials and Methods). “Ia”, “Ib”, or “Ic” indicates a PCR reaction for transcripts initiated from the exon Ia, Ib, or Ic, respectively. The values in the chart denote a percentage of the amount of the *TUBB* transcripts in each cell line. “ND” means “not detected”. (E) The amount of aromatase, which is the product of the *CYP19* gene, was estimated using western blotting analyses. As a loading control, blotting with anti-β-actin was also performed. (F) By using flow cytometry analyses, the homogeneity of the expression of the *CYP19* gene in each cell line was analyzed. Only in JEG-3 cells, a cell population extra-highly expressing the *CYP19* gene was detected (arrow in the panel of JEG-3). The open or the filled gray histogram denotes cell populations stained with mouse IgG isotype control or anti-aromatase antibody, respectively.

The human *CYP19* gene has multiple promoters; three of which are well-characterized. These three promoters—Ia, Ib, and Ic (also known as I.1, I.4, or I.3, respectively)—are located in a region extending about 100 kb upstream of the protein-coding region (Fig 1A). Liver carcinoma-derived HepG2, granulosa cell carcinoma-derived KGN, and placental choriocarcinoma-derived JEG-3 cell lines each express *CYP19* mRNA [28–30]. First, we characterized the usage of each promoter (Ia, Ib, and Ic) in each cell line. Conventional RT-PCR with a separate forward primer for each unique first exon and a reverse primer for the common second exon allowed us to distinguish each of the three distinct transcripts in each line. HepG2 cells expressed transcripts from the Ib and the Ic promoters; KGN cells expressed transcripts only from the Ic promoters; and JEG-3 cells expressed transcripts from all of the promoters (Fig 1B). Note that the Ic transcript in KGN cells was smaller than that in HepG2 and JEG-3 cells because of a distinct splicing site. Human cervical carcinoma–derived HeLa cells were used as a negative control, and as expected, none of the *CPY19* transcripts were evident in these cells.

We next estimated the relative amount of each *CYP19* transcript in each cell line. RT-PCR signals amplified from different primer sets cannot be directly compared among one another because of differences in annealing efficiency of each primer. Instead, the relative amount of each variant was measured following normalization by a standard made from serially diluted human genomic DNA including the same copy number of the sequence for the first exons. Specifically, we performed PCR with primer sets designed to amplify three regions, ones within each first exon (see Materials and Methods). Because HepG2, KGN, and JEG-3 cell lines expressed comparable levels of β-tubulin (*TUBB*) transcripts (Fig 1C and S1 Fig), the relative amount of each *CYP19* transcript was represented as a percentage of the amount of the *TUBB* transcript in a single chart (Fig 1D). In HepG2 cells, transcripts from the Ib promoter were produced at about 0.1% of the *TUBB* control, and transcripts from the Ic promoter were 10-fold less abundant than the Ib transcripts. The level of the Ic transcripts in KGN cells was estimated at approximately 0.001% of the *TUBB* transcripts. Each of the three transcripts in JEG-3 cells was quite abundant, but their amount was not equal; the Ia transcripts were 10% as abundant as the *TUBB* control, but the Ib and the Ic transcripts were each about 0.1% as abundant as the control. As expected, no signal for the variants was detected in HeLa cells. The values measured in such RT-PCR assays seemed to be interpreted as the transcriptional activity of the respective promoters.

We estimated the expression of the *CYP19* gene at the protein level using western blotting analyses. As shown in Fig 1E, JEG-3 cells abundantly produced aromatase, which is encoded by the gene, while a small amount of the protein was detected in HepG2 and KGN cells. As expected, no aromatase was evident in HeLa cells. By using flow cytometry, we analyzed the homogeneity of the expression of the *CYP19* gene in each cell line (Fig 1F). JEG-3 cells showed the existence of subpopulations that highly expressed the *CYP19* gene (arrow in the panel of JEG-3), while a simple peak in the histogram was seen in the other cell lines. Because the pattern of JEG-3 cells was observed even in re-cloned JEG-3 cells (data not shown), the heterogeneity could be an intrinsic property of JEG-3 cells. Therefore, HepG2 and KGN cells were utilized to reveal chromatin structure responsible for the strength of the *CYP19* promoters in further experiments.

### Assessment of chromatin structure in the *CYP19* locus by using the SEVENS assay

**Fig 2:**
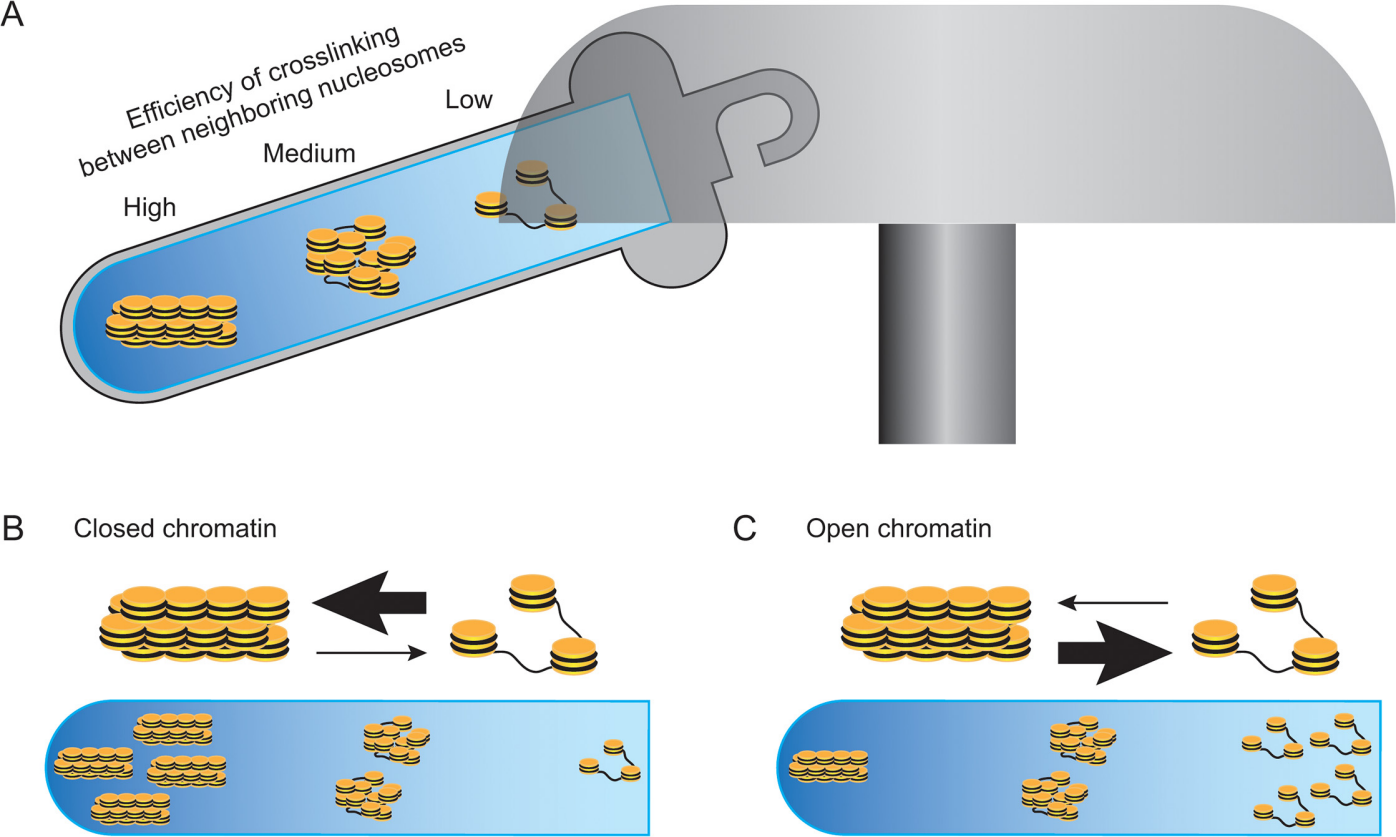
The principle of the SEVENS assay. (A) Neighboring nucleosomes are crosslinked to each other by formaldehyde. Because this crosslinking reagent has a short arm of a single carbon, it preferentially reacts to condensed nucleosomes in closed chromatin. Thus, following solubilization with a detergent and mild sonication, condensed nucleosomes that are sufficiently crosslinked form a large particle, while nucleosomes in open chromatin escape from the crosslinking reaction to be a small particle. When such a preparation is subjected to sedimentation velocity centrifugation with a sucrose density gradient, chromatin is fractionated with regard to the size of sheared particles, namely to the crowdedness of nucleosomes (closed chromatin vs. open chromatin). (B and C) Chromatin structure comprises nucleosomes in equilibrium between a condensed and a dispersed state. Because a preparation for the assay contains multiple fragments of crosslinked chromatin obtained from more than a million cells, data analyzed for a locus-of-interest are represented as the proportion of chromatin graded between open and closed chromatin. Thus, the fractional distribution of a locus in closed chromatin gradually increases toward lower fractions (B). Conversely, a locus in open chromatin increases toward upper fractions (C).

To explore what aspect of chromatin structure determined the strength of the *CYP19* promoters, we used our recently established SEVENS assay [26]. This assay involves the sequential fractionation of formaldehyde-treated chromatin via sedimentation velocity centrifugation and depends on the efficiency of crosslinking between neighboring nucleosomes (Fig 2A). Briefly, closed chromatin consists of closely associated nucleosomes that readily undergo formaldehyde-mediated crosslinking to form larger particles; therefore, closed chromatin quickly sediments into lower fractions (Fig 2B). In contrast, open chromatin with dispersed nucleosomes remains in upper fractions because more of the nucleosomes escape the formaldehyde-mediated crosslinking (Fig 2C). Data for a locus-of-interest is represented as the proportion of chromatin graded from closed to open chromatin, which is obtained from the fractional distribution of the locus in a sucrose density gradient.

**Fig 3:**
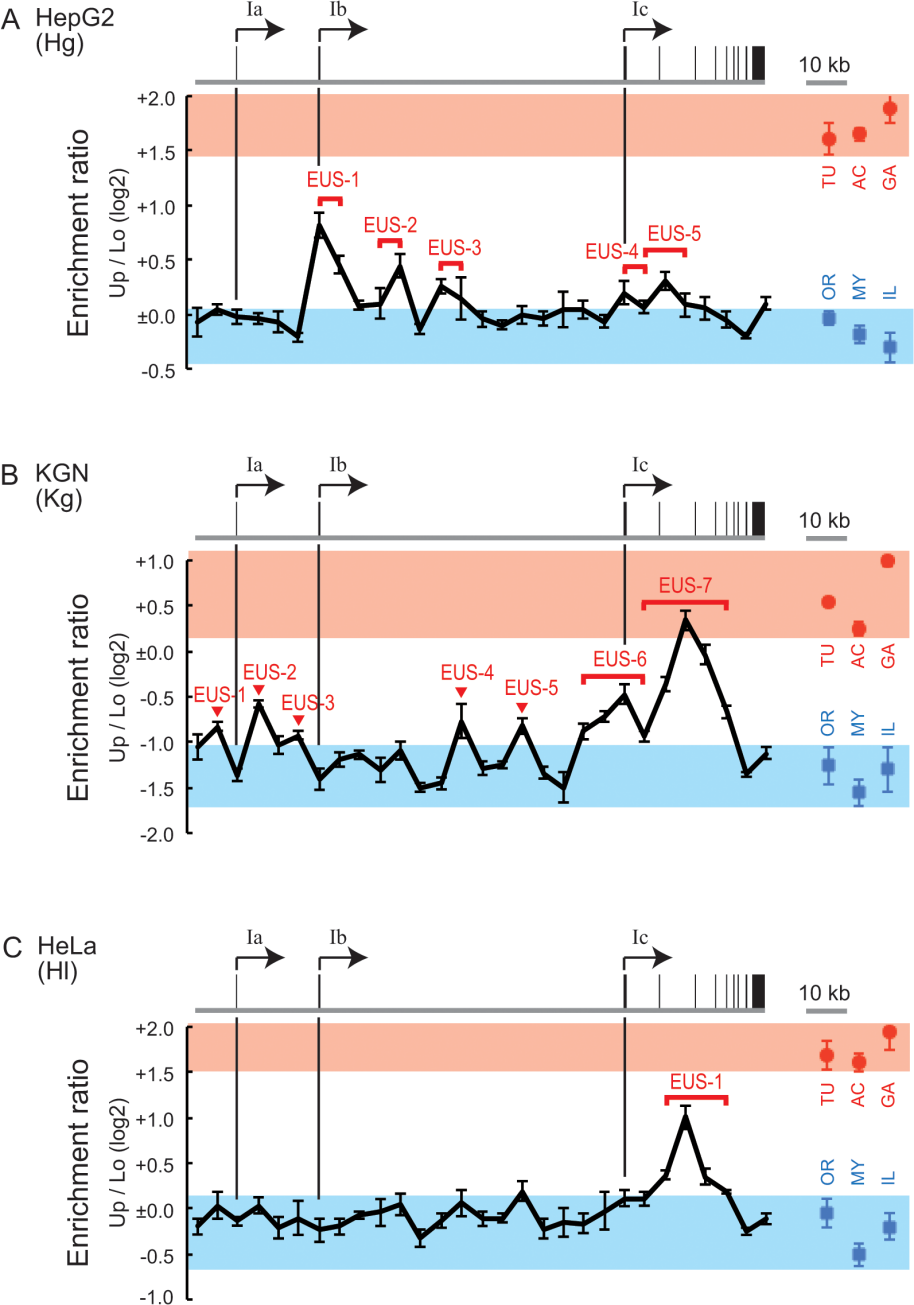
Chromatin structure observed in the SEVENS assay. (A-C) The SEVENS assays revealed distinct chromatin structures in the *CYP19* locus of HepG2 (A), KGN (B), and HeLa cells (C). The proportion of open and closed chromatin is represented as an enrichment ratio of a given sequence in upper fractions to that in lower fractions (Up/Lo) using a log2 scale (see Materials and Methods). The gene structure of the *CYP19* locus is drawn above each chart. Each vertical line extends from a promoter region to note a corresponding position in the respective lower charts. Red circles and blue rectangles denote the Up/Lo values of active and repressed reference loci, respectively, which are abbreviated as TU, AC, GA, OR, MY, or IL for the *TUBB*, *ACTB*, *GAPDH*, *OR1A1*, *MYT1*, or *IL2RA* loci, respectively. To compare assessed regions of the *CYP19* locus to these references, red and blue belts are placed on the charts to indicate the ranges of the Up/Lo values of the references. The positions referred to in the text are labeled as “enrichment in upper fractions of the SEVENS assay (EUS)” in the charts.

**Fig 4:**
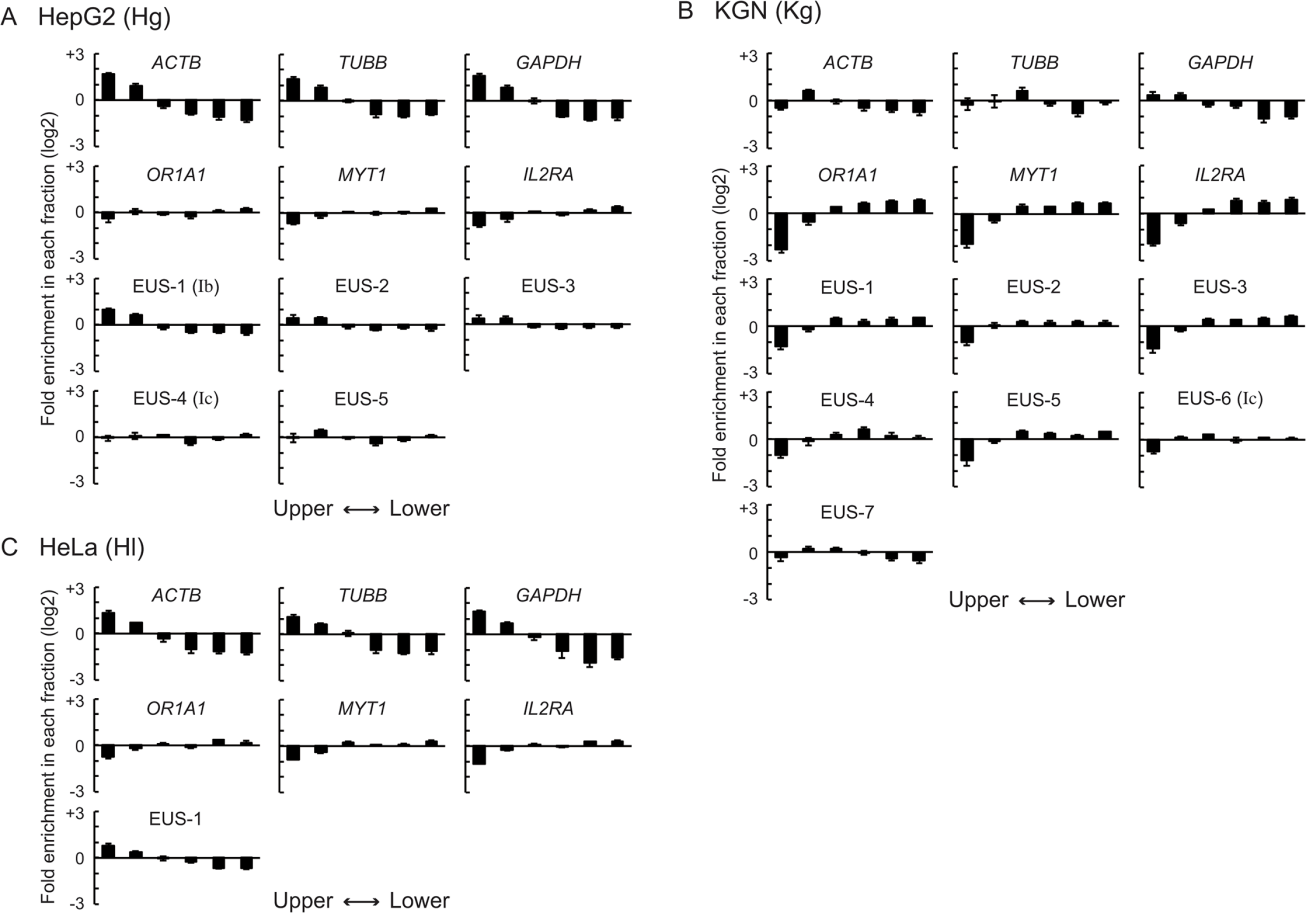
The enrichment of the reference loci and the EUS regions of the *CYP19* locus in individual SEVENS fractions. (A-C) The fractional distribution of the active reference loci (panels in the first row), the repressed reference loci (panels in the second row), and the EUS regions (panels in the third to fifth rows) in HepG2 (A), KGN (B), or HeLa cells (C) was represented as the fold enrichment in each fraction relative to an average calculated from total fractions by using a log2 scale.

The SEVENS assay was performed to examine chromatin structure at six reference loci: *TUBB* (β-tubulin), *ACTB* (β-actin), and *GAPDH* (glyceraldehyde 3-phosphate dehydrogenase) as constitutively active genes; and *OR1A1* (olfactory receptor type 1A1), *MYT1* (myelin transcription factor 1), and *IL2RA* (interleukin 2 receptor α chain) as genes that are repressed in all the cell lines used in this study. The expression profile of these genes was confirmed by RT-PCR (S1 Fig). In the SEVENS assay, the promoter region of the respective loci was assessed, except for *MYT1*, for which a region around the seventh exon was used. The ratio of the abundance of each reference locus in upper fractions to that in lower fractions was calculated (Up/Lo in Fig 3). As expected, the log2 value of the Up/Lo ratio of the three active reference loci was positive, but the three repressed reference loci were negative (red circles and blue rectangles in Fig 3), indicating that at the active reference loci open chromatin was dominant to closed chromatin, while the opposite proportion was found at the repressed reference loci. Notably, the actual values of Up/Lo in HepG2 and HeLa cells were larger than those in KGN cells; for example, the Up/Lo of the *TUBB* gene in HepG2 and HeLa cells was around +1.6, while KGN cells showed +0.5 (TU in Fig 3). To determine the reason for the difference, we also analyzed the enrichment of each reference locus in individual fractions (Fig 4). The active reference loci in HepG2 cells were greatly enriched in the two upper fractions and excluded from the three lower fractions; although the repressed reference loci were excluded from the uppermost fraction, the concentration in the other fractions was close to the average (upper six panels in Fig 4A). This tendency was also seen in HeLa cells (upper six panels in Fig 4C). KGN cells, however, showed a distinct pattern; the repressed reference loci were apparently excluded from the two upper fractions and enriched in the four lower fractions, but the enrichment of the active reference loci in the upper fractions was not clear, although a decrease in the lower fractions was observed (upper six panels in Fig 4B). These observations suggest that the efficiency of chromatin crosslinking differed among the cell lines even when the same conditions were used; chromatin in HepG2 and HeLa cells was unlikely to be crosslinked enough to sediment the repressed reference loci to lower fractions; in contrast, the poor upper enrichment of the active reference loci in KGN cells could result from undesirable excess crosslinking. However, because the chromatin structure of the active and the repressed reference loci within each cell line was distinguished in the SEVENS assay, these reference loci were useful as internal controls to assess chromatin structure in the *CYP19* locus. PCR performed with primer sets that anneal every 5 kb in the *CYP19* locus were used to further characterize the landscape of chromatin structure at the locus.

We first assessed the *CYP19* locus in HepG2 cells (abbreviated as Hg) by using the SEVENS assay (Fig 3A). The distribution of Up/Lo ratios throughout the *CYP19* locus mostly fell on the upper edge of the blue belt representing the range of the ratios of the repressed reference loci. This finding indicated that a major portion of the locus in HepG2 cells was moderately occupied by closed chromatin like the repressed reference loci. The assay also identified five regions relatively enriched in the upper fractions, which were designated as EUS (enrichment in upper fractions of the SEVENS assay) (Hg-EUS-1 to -5 in Fig 3A). The EUS peaks in the figure did not reach the red belt defined by the ratios of the active reference loci, but were above the blue belt. To further elucidate chromatin structure at these EUS regions, the distribution of the regions in individual fractions was analyzed. Hg-EUS-1, which was shown as the highest peak among the five in Fig 3A, was enriched in the two upper fractions, and excluded from the four lower fractions (Fig 4A). However, the magnitude of the biased distribution of Hg-EUS-1 was less than that of the active reference loci. This appeared to be the reason why the peak of Hg-EUS-1 did not reach the red belt in Fig 3A. Likewise, the upper enrichment of Hg-EUS-2 to -5 was reduced compared to the active reference loci, but still higher than that of the repressed reference loci (Fig 4A). Such fractional distributions indicate an intermediate proportion of open chromatin between the active and the repressed reference loci. Importantly, Hg-EUS-1 and -4 coincided with the position of the Ib and Ic promoters, respectively. Since the proportion of open chromatin at Hg-EUS-1 was more than at Hg-EUS-4, this difference was likely to be reflected in the higher activity of the Ib promoter than the Ic promoter (Fig 1D). In addition, the inactive Ia promoter was plotted within the blue belt, suggesting a large proportion of closed chromatin and a small proportion of open chromatin, like the repressed reference loci. Therefore, the strength of the *CYP19* promoters in HepG2 cells was well reflected in the chromatin proportion (open chromatin vs. closed chromatin) as determined via the SEVENS assay.

When chromatin at the *CYP19* locus in KGN cells (abbreviated as Kg) was examined in the SEVENS assay, regions with a relatively high Up/Lo ratio were evident at Kg-EUS-1 to -7 (Fig 3B). Because the Up/Lo ratio at Kg-EUS-7 reaches the red belt in the figure, chromatin structure at this region appeared to be mostly open like the active reference loci. In fact, the panel of Kg-EUS-7 in Fig 4B shows a similar fractional distribution to that of the active reference loci, in which Kg-EUS-7 was excluded from the two lower fractions although the upper enrichment was not clear. The Up/Lo ratios at the other EUS peaks fell between the blue and the red belts (Fig 3B), suggesting that these regions had an intermediate proportion of open and closed chromatin. Interestingly, one peak, Kg-EUS-6, coincided with the position of the Ic promoter, which is the only active promoter in KGN cells. This intermediate proportion seemed to be required for the active state of the Ic promoter even though its activity was quite weak (Fig 1D). Notably, the regions of the Ia and the Ib promoters fell into the blue belt in Fig 3B, indicating that they were both mostly occupied by closed chromatin like the repressed reference loci. Thus, the absence of open chromatin could be responsible for the repressed state of these promoters.

Finally, chromatin structure in HeLa cells (abbreviated as Hl) was also analyzed in the SEVENS assay (Fig 3C). A single region with a high Up/Lo ratio was observed and labeled as Hl-EUS-1. When the fractional distribution of this region was analyzed, a moderate enrichment in upper fractions accompanied by a moderate exclusion from lower fractions was observed (Fig 4C). This observation indicates that the proportion of open chromatin at Hl-EUS-1 was relatively large, but less than that of the active reference loci. Importantly, the Up/Lo ratio in the upstream three-quarters of the *CYP19* locus in HeLa cells fell almost entirely in the blue belt (Fig 3C). A high proportion of closed chromatin, which was also interpreted as a low proportion of open chromatin, appeared to contribute to the repression of the *CYP19* promoters in HeLa cells.

### Relationship between nucleosome occupancy and chromatin structure observed in the SEVENS assays

**Fig 5:**
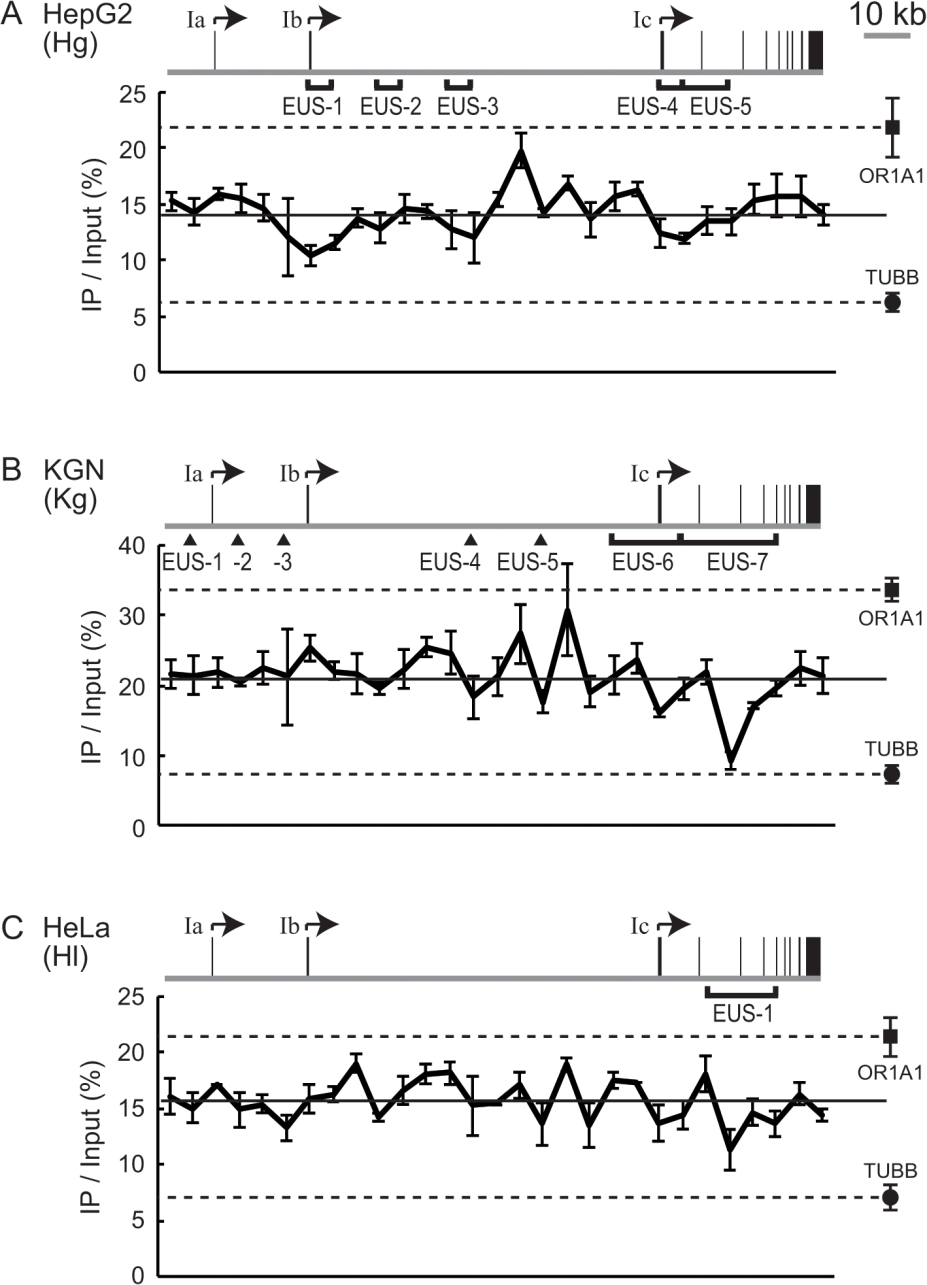
The nucleosome occupancy in the *CYP19* locus. (A-C) ChIP assays with anti-pan-histone H3 revealed the amount of nucleosomes in the *CYP19* locus in HepG2 (A), KGN (B), or HeLa cells (C). The nucleosome level is expressed as a percentage of total input chromatin (IP/Input). The solid line in the respective charts represents the average value of IP/Input. The level at the *OR1A1* (■) or the *TUBB* (●) locus as a reference is also plotted at the right of each chart. To compare a given region in the *CYP19* locus to the references, broken lines are drawn from the reference marks. The gene structure of *CYP19* and the EUS regions are also represented on each chart.

Nucleosomes are omitted from the promoter region of a highly expressed gene; such promoter regions are known as nucleosome-depleted regions (NDRs) [10–12]. To assess whether the EUS regions indicated by the SEVENS assay were correlated to a low occupancy of nucleosomes, we performed ChIP assays with a pan-antibody that recognizes modified and unmodified histone H3. We first examined nucleosomes at the *CYP19* locus in HepG2 cells (Fig 5A). Three regions that corresponded to Hg-EUS-1, -3, and -4 were observed with relatively lower nucleosome occupancy. Because the occupancy at these regions was still above the background level seen at the NDR in the *TUBB* promoter, in a subpopulation of the cells, nucleosomes appeared to be removed from the regions. Since Hg-EUS-1 and -4 coincided with the position of the Ib and the Ic promoters, respectively, the partial removal of nucleosomes could be reflected in the occurrence of EUS and eventually in the moderate activity of these promoters.

When KGN cells were subjected to the ChIP assay, four regions with low nucleosome occupancy were evident and coincided with Kg-EUS-4 to -7 (Fig 5B). Of these regions, only Kg-EUS-7 had a similar level to that at the NDR of the *TUBB* promoter, suggesting that open chromatin seen at Kg-EUS-7 seems to be due to the absence of nucleosomes. Because occupancy at the other regions did not fall down to the *TUBB* level, nucleosomes appeared to be removed in some of the cells. Such nucleosome removal could result in a moderate proportion of open chromatin at the EUS regions, particularly at Kg-EUS-6, in a weak but significant activity of the Ic promoter.

When the nucleosome occupancy of HeLa cells was analyzed, one region with a relatively low level of nucleosomes was observed and coincided with Hl-EUS-1 (Fig 5C). This seemed to be responsible for a large proportion of open chromatin at Hl-EUS-1.

Altogether, all the regions with low nucleosome occupancy were mapped to the positions of the EUS regions. Therefore, we confirmed that the proportion of nucleosome-free chromatin was directly reflected in the proportion of open chromatin judged in the SEVENS assay. However, some EUS regions were not identified as regions with a low level of nucleosomes; for example, no particular feature in nucleosome occupancy corresponded to Hg-EUS-2 or Kg-EUS-2. These discrepancies may result from the different spatial resolution achieved in an entire sheared sample in the ChIP assays vs. that achieved with a size-fractionated sample in the SEVENS assays.

### The distribution of tri-methylation and acetylation of histone H3 lysine 27 in the *CYP19* locus

Histone H3 tri-methylated at lysine 27 (H3K27me3) is an epigenetic mark related to gene silencing, and it also accumulates at promoters with low activity [31]. In addition, acetylation of the same lysine residue of histone H3 (H3K27ac) is correlated to the amount of transcripts from promoters containing a CpG island [32]. Here, to assess the relationship between these epigenetic marks and structural variation related to promoter activity of the *CYP19* gene, we examined the distribution of H3K27me3 and H3K27ac throughout the locus by using ChIP assays with an antibody that recognizes each epigenetic mark. Because the amount of nucleosomes at each assessed position of the *CYP19* locus was not equal as shown in Fig 5, the level of the precipitations was normalized to total histone H3 to be represented as relative occupancy of the modifications.

**Fig 6:**
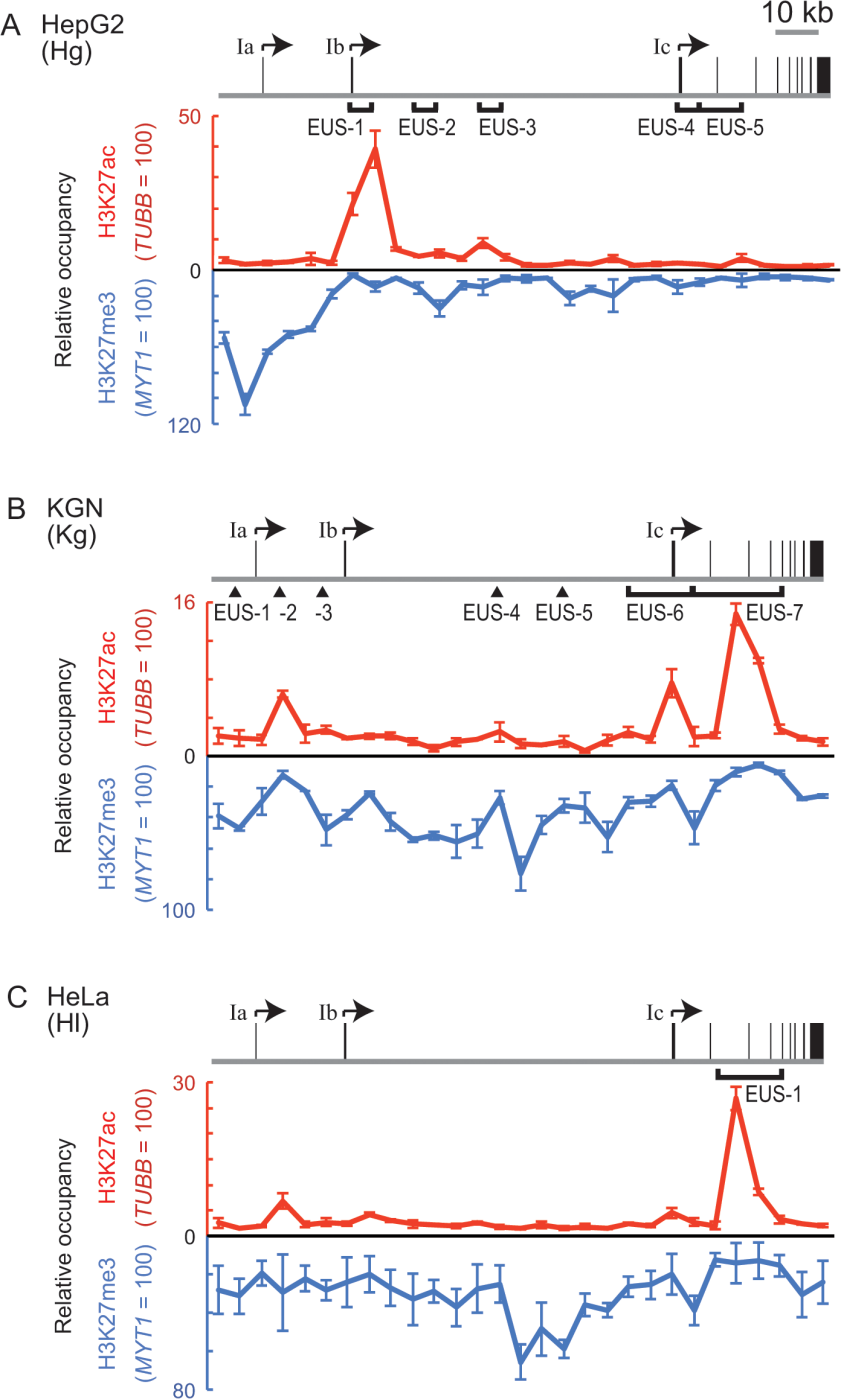
The distribution of H3K27me3 and H3K27ac in the *CYP19* locus. (A-C) ChIP assays with anti-H3K27me3 (blue lines) or anti-H3K27ac (red lines) revealed the distribution of these histone modifications in the *CYP19* locus in HepG2 (A), KGN (B), or HeLa cells (C). Following normalization to the amount of total histone H3, relative occupancy of H3K27me3 or H3K27ac is represented by using a percentage of the modification at the *MYT1* or the *TUBB* control locus, respectively. Note that the direction of the y-axis of the charts for H3K27me3 is downward. The gene structures of *CYP19* and the EUS regions are also represented on each chart.

We first examined the distribution of H3K27me3 in HepG2 cells (blue line in Fig 6A). An apparent enrichment of the tri-methylation was evident in the most upstream 20-kb region. The enrichment peaked 5 kb upstream of the Ia promoter and reached the level of the *MYT1* control locus, which is a target gene of a polycomb repressive complex [33]. This high level of tri-methylation probably contributes to the proportion of closed chromatin represented in the blue belt of Fig 3A. Importantly, this modification extended over the Ia promoter, suggesting that the inactive state of the promoter could be due to closed chromatin containing H3K27me3. Downstream of the region, the abundance of H3K27me3 decreased and remained close to the background level. The ChIP assay with anti-H3K27ac revealed a single region where the acetylation was enriched, which was distributed over the Ib promoter (red line in Fig 6A). The acetylation could be responsible for the large proportion of open chromatin evident at Hg-EUS-1 and for the activity of the Ib promoter in HepG2 cells.

KGN cells were subjected to the ChIP assays with anti-H3K27me3 and anti-H3K27ac. An intermediate level of H3K27me3 was evident throughout the *CYP19* locus of KGN cells (blue line in Fig 6B). In this distribution, the level of H3K27me3 at Kg-EUS-2 and -7 was relatively decreased, suggesting that H3K27me3 was negatively correlated to the proportion of open chromatin. When H3K27ac was examined, three apparent peaks were evident and colocalized with Kg-EUS-2, -6, and -7 (red line in Fig 6B). In particular, the highest level of the acetylation was seen at Kg-EUS-7, suggesting that the abundant acetylation was probably responsible for completely open chromatin observed in the SEVENS assay. Thus, in addition to the absence of H3K27me3, the presence of H3K27ac seemed to enhance the frequency of open chromatin. One of the acetylated regions was mapped on the Ic promoter; although its activity was quite low (Fig 1D), the acetylation appeared to be required for transcription from the Ic promoter that was embedded in Kg-EUS-6.

We also performed the ChIP assays for HeLa cells. In addition to a moderate level of H3K27me3 throughout the *CYP19* locus, a region between the Ib and the Ic promoters was relatively highly H3K27-tri-methylated (blue line in Fig 6C). Although H3K27ac was barely evident in most regions of the locus, the acetylation accumulated at the region of Hl-EUS-1 (red line in Fig 6C). This high level of H3K27ac could be reflected in the structure of Hl-EUS-1.

### Ectopic transcription from the Ia promoter of the *CYP19* gene in HepG2 cells treated with an H3K27me3-inhibitor

**Fig 7:**
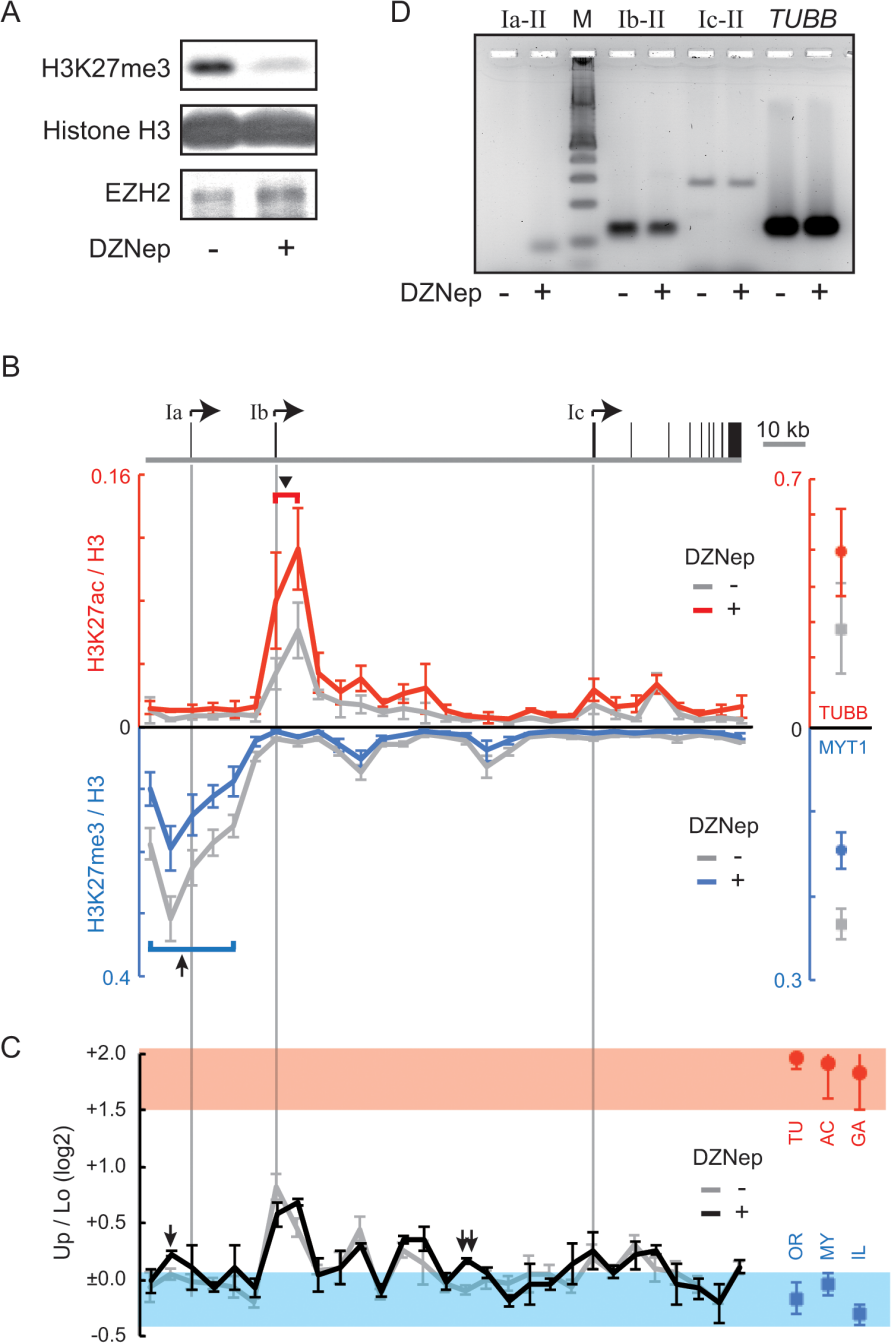
Ectopic transcription from the Ia promoter in DZNep-treated HepG2 cells. (A) Western blotting analyses were used to assess the effect of DZNep on the level of H3K27me3 in HepG2 cells. As loading controls, blotting signals from total histone H3 and a histone methyltransferase EZH2 were also monitored. The experiments with the lysate from DZNep-treated or untreated HepG2 cells are denoted as “+” or “-”, respectively. (B) ChIP assays were used to assess the distribution of H3K27me3 (blue line) or H3K27ac (red line) in DZNep-treated HepG2 cells. As controls, the level of H3K27me3 at the *MYT1* locus or H3K27ac at the *TUBB* locus is also shown at the right of each panel. The results obtained from untreated HepG2 cells are drawn in gray. ChIP values are normalized to the amount of total histone H3 because of the variation in nucleosome occupancy. The direction of the y-axis of the chart for H3K27me3 is downward. To indicate the position of the *CYP19* promoters, each gray line extends from the gene structure drawn above the charts. An arrow or an arrowhead in the charts marks the positions referred to in the text. (C) The SEVENS assays revealed chromatin structure in the *CYP19* locus in DZNep-treated HepG2 cells (black line). The data of untreated cells (referred from Fig 3A) is also represented in the chart (gray line). The structure at the reference loci in DZNep-treated HepG2 cells are shown at the right of the panel, in which the *TUBB*, *ACTB*, *GAPDH*, *OR1A1*, *MYT1*, and *IL2RA* loci are abbreviated as TU, AC, GA, OR, MY, and IL, respectively. Red and blue belts are also indicated on the chart to show the range of the references. The Up/Lo value at the position marked by an arrow or a double arrow in DZNep-treated cells is significantly larger than that in untreated cells (both p < 0.05). (D) Conventional RT-PCR analyses were performed to check the existence of the *CYP19* transcripts in DZNep-treated (+) or untreated (-) HepG2 cells. The keys (“Ia-II”, “Ib-II”, or “Ic-II”) denote a PCR reaction performed with a primer set for the transcripts produced from the Ia, the Ib, or the Ic promoter, respectively. Additionally, control experiments were performed with a primer set for the *TUBB* gene. “M” denotes a molecular weight marker.

To verify that the proportion of open chromatin at the *CYP19* promoters was linked to their activity, we designed an experiment involving manipulation of the chromatin structure. Our analysis of HepG2 cells suggested that enrichment of H3K27me3 around the Ia promoter contributed to formation of the closed chromatin that extended over the Ia promoter (Figs 3A and 6A). Here, we used 3-deazaneplanocin A (DZNep) to block the introduction of H3K27me3 [34]. DZNep treatment was expected to eventually change the Up/Lo ratio of the Ia promoter in HepG2 cells. To assess the effect of DZNep on HepG2 cells, we performed western blot analyses (Fig 7A). H3K27me3 was dramatically impaired by DZNep treatment, although the total amount of histone H3 was not affected. DZNep did not greatly affect the expression of the methylating enzyme EZH2, as was shown previously [34]. The distribution of H3K27me3 in the *CYP19* locus was analyzed in ChIP assays (blue line in Fig 7B). The treatment with DZNep reduced the enrichment of H3K27me3 around the Ia promoter, but H3K27me3 levels around the promoter were still higher than those in the downstream regions (arrow in Fig 7B). Because H3K27me3 at the *MYT1* reference locus was also reduced (right panel in Fig 7B), DZNep may impair methylation throughout the genome. Interestingly, the distribution of H3K27ac was also affected by DZNep treatment (red line in Fig 7B); the acetylation around the Ib promoter region was increased by the treatment (arrowhead in Fig 7B). DZNep treatment also increased the level of H3K27ac at the *TUBB* reference locus (right panel in Fig 7B); therefore, DZNep seemed to cause additional acetylation in regions where H3K27ac had already accumulated.

The effect of DZNep on the proportion of open and closed chromatin in HepG2 cells was verified by using the SEVENS assay (Fig 7C). DZNep treatment did not significantly affect chromatin structure of the reference loci; the Up/Lo ratios of the active reference loci remained higher than +1.5, while those of the repressed reference loci remained less than 0 (red circles and blue rectangles in Fig 7C). Similarly, Up/Lo ratios throughout the *CYP19* locus did not appear to be changed following treatment with DZNep (black vs. gray line in Fig 7C). However, when statistical analyses were performed to compare the Up/Lo values at each position between DZNep-treated and untreated cells, the Up/Lo ratio was significantly increased at only two positions (arrow and double arrow in Fig 7C; both p < 0.05). Because the Up/Lo peak at these positions moved out of the blue belt that represented the chromatin proportion of the repressed reference loci, the amount of closed chromatin was reduced, and the proportion of open chromatin increased. Importantly, the position marked by the arrow was just upstream of the Ia promoter.

Finally, to confirm the existence of the *CYP19* transcripts produced from the Ia promoter in HepG2 cells, we performed a conventional RT-PCR analysis with a primer set targeting the Ia exon and the common second exon. As shown in the Ia-II lanes of Fig 7D, a PCR amplicon derived from the transcripts containing the exon Ia was detected in the DZNep-treated cells, but not in untreated cells. The expression level of the ectopic transcripts, however, was unquantifiable because of the amount close to an experimental background. This finding indicated that slight but meaningful transcription occurred from the Ia promoter, which was close to a region where open chromatin appeared artificially. In contrast, the amount of the *CYP19* transcripts produced from the Ib and Ic promoters and of the *TUBB* transcripts was unaffected by treatment with DZNep (Fig 7D).

## Discussion

For several decades, endonucleases such as DNase I have been widely used to assess chromatin structure, particularly nucleosome positioning, in the genome [35]. When native chromatin is treated with such an enzyme, regions where the DNA is digested are identified as open and accessible to the enzyme. Another assay for chromatin structure, called FAIRE, can concentrate a protein-free and open region of chromatin into an aqueous phase following extraction with phenol [36]. Thus, these methods are specialized for detection of open chromatin. Instead, we have recently succeeded in developing a sequential fractionation method based on sedimentation velocity centrifugation (Fig 2). This assay, which is designated as the SEVENS assay, can distinguish chromatin that are graded from open to closed structures [26]. Because sheared chromatin obtained from more than a million cells is used as starting materials in the assay, data represent the proportion of graded structures in a mixture of multiple chromatin fragments. Intriguingly, when the promoter region of the active reference loci, which is expected to comprise open chromatin, was fractionated, the distribution was gradually increased from lower to upper fractions (panels in the first row of Fig 4A–4C). Similarly, although the repressed reference loci are usually embedded in closed chromatin, their fractional distribution was gradually increased from upper to lower fractions (panels in the second row of Fig 4A–4C). The presence of these graded distributions implies that chromatin, referred to as a typically open or a typically closed structure, may not be stable, but rather may tend toward an open or a closed state, respectively, in equilibrium between the two opposite structures. This idea is supported by a previous report that the interaction between neighboring nucleosomes is so dynamic that chromatin structure is determined by equilibrium between dispersed and condensed states of nucleosomes [37]. An *in vivo* imaging analysis has also revealed continuous fluctuation of nucleosomes in interphase chromatin [38]. Therefore, even when the promoter of the active reference loci was examined, a small proportion of closed as well as intermediate chromatin was detected in a snapshot of crosslinked chromatin. Conversely, a small part of closed chromatin at the repressed reference loci appeared to be distributed as open or intermediate chromatin in the SEVENS fractionation.

To assess chromatin structure in the *CYP19* locus, the SEVENS assay was utilized in this study. Many of the assessed positions in the locus showed a low Up/Lo ratio similar to that at the repressed reference loci (blue belts in Fig 3). This finding indicates that a large ortion of the *CYP19* locus was occupied by closed chromatin like at the repressed reference loci. On the other hand, some regions with a relatively high Up/Lo ratio were identified and designated as the EUS regions (Fig 3). Local recruitment of DNA-binding proteins such as TFs and secondary proteins that often drive chromatin remodeling could cause the occurrence of EUS. Importantly, the values of the Up/Lo ratios differed among the EUS regions; they did not reach the Up/Lo ratios at the active reference loci, except for at Kg-EUS-7. These moderate Up/Lo ratios were due to an intermediate tendency of the fractional distribution between the active and the repressed references, which was observed as a moderate proportion of open and closed chromatin (Fig 4). Note that any EUS regions were not enriched in central fractions. In general, open chromatin is believed to be nucleosome-free chromatin. Many of the EUS regions were also identified as regions with a low occupancy of nucleosomes (Figs 3 vs. 5). Therefore, when removal of nucleosomes is more frequent than deposit of nucleosomes in chromatin structure in equilibrium, the EUS region could appear. Five EUS regions with higher Up/Lo ratios, Hg-EUS-1, Kg-EUS-2, Kg-EUS-6, Kg-EUS-7, and Hl-EUS-1, coincided with locations of H3K27ac (Fig 3 vs. red lines in Fig 6). This correlation suggests that H3K27ac is likely to contribute to enhancing the frequency of nucleosome removal via a decrease in the affinity between neighboring nucleosomes [15]. In contrast, a repressive epigenetic mark H3K27me3 may not always be required for nucleosome deposit, because some regions with the Up/Lo ratio plotted in the range of the repressed references were observed independent of the recruitment of the mark (Fig 3 vs. blue line in Fig 6).

The active *CYP19* promoters, the Ib and the Ic promoters in HepG2 cells and the Ic promoter in KGN cells, coincided with the EUS regions, Hg-EUS-1, Hg-EUS-4, and Kg-EUS-6, respectively, while none of the inactive promoters coincided with such regions (Fig 3). Ectopic transcription also occurred at the Ia promoter of DZNep-treated HepG2 cells, which is close to the region with an increased proportion of open chromatin (Fig 7C and 7D). These findings indicate that activity of the *CYP19* promoters is likely to require a shift toward open chromatin in the equilibrium between closed and open chromatin. The proportion of open chromatin in Hg-EUS-1 was larger than in Hg-EUS-4 (Fig 4A), suggesting that nucleosome removal at the Ib promoter could be more frequent than at the Ic promoter. The difference in the frequency is likely to result in the activity of the Ib promoter being 10-fold stronger than that of the Ic promoter in HepG2 cells (Fig 1D).

In general, the transcriptional level of active genes varies; housekeeping genes usually produce a large amount of transcripts, whereas the amount of RNA from some of the tissue-specific genes appears to be restricted. This variation is thought to come from transcription at an appropriate level of individual genes. A genome-wide SEVENS analysis will give us hints as to how transcription is quantitatively controlled.

## Supporting Information

S1 Table PCR primers for RT-PCR, SEVENS, and ChIP assays. The primers for RT-PCR are designed for the exons shown in parentheses. To represent the position of the primers for SEVENS and ChIP assays, the distance from the TSS (the Ia TSS for the *CYP19* gene) is included in parentheses.
(PDF)

S1 Fig Expression profiles of reference genes in HepG2, KGN, and HeLa cells. RT-PCR analyses were performed using 100 pg of cDNA as a template, which was synthesized from total RNA purified from either of the cell lines. The PCR reactions with 100 pg of genomic DNA (DNA from human placenta: #D3035, Sigma-Aldrich) were also performed as a positive control (designated as “Control”). Therefore primer sets that amplify a region encoded by a single exon of the respective genes were utilized. The primers used in this study are listed in S1 Table.
(PDF)
