# Supplementary material for: The Proportion of Chromatin Graded between Closed and Open States Determines the Level of Transcripts Derived from Distinct Promoters in the CYP19 Gene
Source: PLoS One. 2015 May 28;10(5):e0128282. doi: 10.1371/journal.pone.0128282 (PMC4447357; doi:10.1371/journal.pone.0128282)
Supplement: S1 Table — The primers for RT-PCR are designed for the exons shown in parentheses. To represent the position of the primers for SEVENS and ChIP assays, the distance from the TSS (the Ia TSS for the CYP19 gene) is included in parentheses. (PDF) [file pone.0128282.s001.pdf]

S1 Table. PCR primers for RT-PCR, SEVENS, and ChIP assays.

Usage	Figure	Gene	Forward primer	Reverse primer
Conventional RT-PCR	Figs. 1B & 7D	*TUBB* (exon I - exon II)	CATACCTTGAGGCGAGCAAA	CGATGCCATGTTCATCACTG
Conventional RT-PCR	Figs. 1B & 7D	*CYP19* (exon Ia - exon II)	TGCTCGGGATCTTCCAGAC	GTATCGGGTTCAGCATTTCC
Conventional RT-PCR	Figs. 1B & 7D	*CYP19* (exon Ib - exon II)	TGACCAACTGGAGCCTGACA	GTATCGGGTTCAGCATTTCC
Conventional RT-PCR	Figs. 1B & 7D	*CYP19* (exon Ic - exon II)	CTTGGGCTTCCTTGTTTTGA	GTATCGGGTTCAGCATTTCC
Conventional RT-PCR	S1 Fig.	*TUBB* (exon I)	CATTCCAACCTTCCAGCCTG	TTTGCTCGCCTCAAGGTATG
Conventional RT-PCR	S1 Fig.	*ACTB* (exon III)	TGAAGTACCCCATCGAGCAC	CACACGCAGCTCATTGTAGA
Conventional RT-PCR	S1 Fig.	*GAPDH* (exon IX)	CAGCAAGAGCACAAGAGGAA	CTACATGGCAACTGTGAGGA
Conventional RT-PCR	S1 Fig.	*OR1A1* (exon I)	TCAGAAGTGATGGAGAAGGA	TGGTTATTTTCCCTCATGGC
Conventional RT-PCR	S1 Fig.	*MYT1* (exon VII)	CCGTCAAGTCCCATTTTGGA	AGTTCAGGAGAGAAGTTGCG
Conventional RT-PCR	S1 Fig.	*IL2RA* (exon II)	CCTACAAGGAAGGAACCATG	GAGTGGCTAGAGTTTCCTGT
Quantitative RT-PCR	Fig. 1C	18S rDNA	AAACGGCTACCACATCCAAG	ATTCCAATTACAGGGCCTCG
Quantitative RT-PCR	Figs. 1C & 1D	*TUBB* (exon I)	CATTCCAACCTTCCAGCCTG	TTTGCTCGCCTCAAGGTATG
Quantitative RT-PCR	Fig. 1D	*CYP19* (exon Ia)	ACGTGGAGGCAAACAGGAAG	GTCTGGAAGATCCCGAGCA
Quantitative RT-PCR	Fig. 1D	*CYP19* (exon Ib)	AAAGTGTCCTTGATCCCAGG	TAGTCTTGGTTGGTCATGGG
Quantitative RT-PCR	Fig. 1D	*CYP19* (exon Ic)	AGGTTCTATCAGACCAAGCG	TCAAAACAAGGAAGCCCAAG
SEVENS & ChIP	Figs. 3-7	*TUBB* (±0 kb)	CATTCCAACCTTCCAGCCTG	TTTGCTCGCCTCAAGGTATG
SEVENS & ChIP	Figs. 3-7	*ACTB* (±0 kb)	TGCGCCGTTCCGAAAGTTG	AAAGGCGAGGCTCTGTGCT
SEVENS & ChIP	Figs. 3-7	*GAPDH* (±0 kb)	GCTACTAGCGGTTTTACGGG	CTGCGGGCTCAATTTATAGA
SEVENS & ChIP	Figs. 3-7	*OR1A1* (±0 kb)	TCAGAAGTGATGGAGAAGGA	TGGTTATTTTCCCTCATGGC
SEVENS & ChIP	Figs. 3-7	*MYT1* (+43 kb)	CCGTCAAGTCCCATTTTGGA	AGTTCAGGAGAGAAGTTGCG

SEVENS & ChIP	Figs. 3-7	*IL2RA* (±0 kb)	TGAGATGAGAGAAGAGAGTG	TCCATCCAGTCTCTATCGGA
SEVENS & ChIP	Figs. 3-7	*CYP19* (-10 kb)	TCCTGAGTATCTGGGACAAG	CAGGCTAGACACAGTGGTTC
SEVENS & ChIP	Figs. 3-7	*CYP19* (-5 kb)	TCCGAATTGCAACCTATTCC	CAGAGTTAGTGGTAGCACTG
SEVENS & ChIP	Figs. 3-7	*CYP19* (±0 kb), the Ia promoter	TTTGCCCTCCTTTCATCCAC	ATTCCTTCCTCCAGGGTATG
SEVENS & ChIP	Figs. 3-7	*CYP19* (+5 kb)	CTGTGGACCCTAAACATGTG	GATACTGGTGCCCTCTTATG
SEVENS & ChIP	Figs. 3-7	*CYP19* (+10 kb)	AACTCAGGATTCCCAGTGAG	CCACATTCACCGTCGTAACA
SEVENS & ChIP	Figs. 3-7	*CYP19* (+15 kb)	GACACTGAATTTCACAGTGG	GGATTTGGATGTGTCTCTAC
SEVENS & ChIP	Figs. 3-7	*CYP19* (+20 kb), the Ib promoter	GGTTCATCTGTCGTCTTCAG	GAACCACATATTTCCCCAAG
SEVENS & ChIP	Figs. 3-7	*CYP19* (+25 kb)	CCCAAAGTGCTGGGATTACA	AGAGCCCTTGCTCACGTTTG
SEVENS & ChIP	Figs. 3-7	*CYP19* (+30 kb)	TTGGGGTCTGAATATGTACC	TCCTTTCCCCAAACTCCTCC
SEVENS & ChIP	Figs. 3-7	*CYP19* (+35 kb)	TGACTTTCAGAAGCTCTGCC	ATACACACAGCCCAGGCTAA
SEVENS & ChIP	Figs. 3-7	*CYP19* (+40 kb)	CATCTAGTCTGCTTTTTCCC	CCACCTCTAGATCAGCTAAG
SEVENS & ChIP	Figs. 3-7	*CYP19* (+45 kb)	TTGGATCGGCATCAGAGGGA	CCTTGATGCCAAAGAATGCC
SEVENS & ChIP	Figs. 3-7	*CYP19* (+50 kb)	CAAAGGGTGAGAGGATGGAG	TCTTGCACCTCAGGTTTCAC
SEVENS & ChIP	Figs. 3-7	*CYP19* (+55 kb)	CATAGCCTGAGCTGAAAGAG	TCAAGCAAGCCATTACTCTG
SEVENS & ChIP	Figs. 3-7	*CYP19* (+60 kb)	ACTGTAAAGTAGCCCCACAA	CATGCACACACATAGATACC
SEVENS & ChIP	Figs. 3-7	*CYP19* (+65 kb)	TCCTAAGAAGAGGGTGAGAG	TCATAGTTTTGGAGTGGCAG
SEVENS & ChIP	Figs. 3-7	*CYP19* (+70 kb)	ATCCATCATAGCCTTGCTTG	AGCTATGCCACAGTTTACCC
SEVENS & ChIP	Figs. 3-7	*CYP19* (+75 kb)	ATGCAAGAATCCACCCTCAC	GAGAATAGACAGAATGGGTG
SEVENS & ChIP	Figs. 3-7	*CYP19* (+80 kb)	TAGTAGGAGTCCTGCTGGAA	ATTCCAACAAAGCCCAAGTG
SEVENS & ChIP	Figs. 3-7	*CYP19* (+85 kb)	AGGTGGATAGAAAATGTGGG	AGAGCCACGAGGTCACTGAA
SEVENS & ChIP	Figs. 3-7	*CYP19* (+90 kb)	CTCTGTAAGAGGTGTGAGGA	GCCATAGGGAACTCACCATT
SEVENS & ChIP	Figs. 3-7	*CYP19* (+95 kb), the Ic promoter	GACCTCAACGATGCCCAAGA	AAAGGCAATCTCCCAACTCC

SEVENS & ChIP	Figs. 3-7	*CYP19* (+100 kb)	CACGGCAGATTCCTGTGGAT	CTCTCCAGAGATCCAGACTC
SEVENS & ChIP	Figs. 3-7	*CYP19* (+105 kb)	ACAGGGGCTTCCTTCATTTA	GGATGAAGAGCCAATAATGAC
SEVENS & ChIP	Figs. 3-7	*CYP19* (+110 kb)	AGCTTCCTTTACTACCACAG	GAGTATCAGTCAGGGCTTGA
SEVENS & ChIP	Figs. 3-7	*CYP19* (+115 kb)	GGATAGTCTTCTTCTGGAGTT	CTTAAAGTGCCCCTTTCTCT
SEVENS & ChIP	Figs. 3-7	*CYP19* (+120 kb)	TACAGAAAGTGCTATCGTGG	ACTTACACAGACTTCTCATAC
SEVENS & ChIP	Figs. 3-7	*CYP19* (+125 kb)	ACTACTTCCTAGTGAGCACC	TTGGCACACTCAAGGGTCTA
SEVENS & ChIP	Figs. 3-7	*CYP19* (+130 kb)	TTCAGTAGGCTGATGAACAC	GCACATTGCACATTCAAGAG
