# Supplementary figures and images for: The Proportion of Chromatin Graded between Closed and Open States Determines the Level of Transcripts Derived from Distinct Promoters in the CYP19 Gene
Source: PLoS One. 2015 May 28;10(5):e0128282. doi: 10.1371/journal.pone.0128282 (PMC4447357; doi:10.1371/journal.pone.0128282)

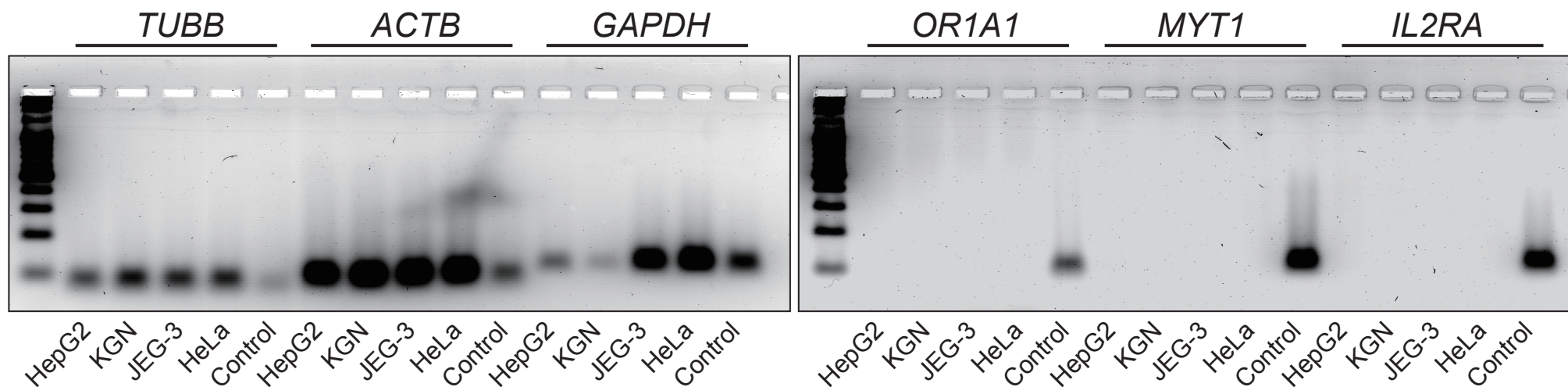

S1 Fig. Expression profiles of reference genes in HepG2, KGN, JEG-3 and HeLa cells.

Supplement: S1 Fig — RT-PCR analyses were performed using 100 pg of cDNA as a template, which was synthesized from total RNA purified from either of the cell lines. The PCR reactions with 100 pg of genomic DNA (DNA from human placenta: #D3035, Sigma-Aldrich) were also performed as a positive control (designated as “Control”). Therefore primer sets that amplify a region encoded by a single exon of the respective genes were utilized. The primers used in this study are listed in S1 Table. (PDF) [file pone.0128282.s002.pdf]
